# The Chromatin Protein DUET/MMD1 Controls Expression of the Meiotic Gene *TDM1* during Male Meiosis in *Arabidopsis*

**DOI:** 10.1371/journal.pgen.1005396

**Published:** 2015-09-08

**Authors:** Sébastien Andreuzza, Bindu Nishal, Aparna Singh, Imran Siddiqi

**Affiliations:** Centre for Cellular and Molecular Biology (CSIR), Hyderabad, India; INRA, FRANCE

## Abstract

Meiosis produces haploid cells essential for sexual reproduction. In yeast, entry into meiosis activates transcription factors which trigger a transcriptional cascade that results in sequential co-expression of early, middle and late meiotic genes. However, these factors are not conserved, and the factors and regulatory mechanisms that ensure proper meiotic gene expression in multicellular eukaryotes are poorly understood. Here, we report that DUET/MMD1, a PHD finger protein essential for *Arabidopsis* male meiosis, functions as a transcriptional regulator in plant meiosis. We find that DUET-PHD binds H3K4me2 in vitro, and show that this interaction is critical for function during meiosis. We also show that DUET is required for proper microtubule organization during meiosis II, independently of its function in meiosis I. Remarkably, DUET protein shows stage-specific expression, confined to diplotene. We identify two genes *TDM1* and *JAS* with critical functions in cell cycle transitions and spindle organization in male meiosis, as DUET targets, with *TDM1* being a direct target. Thus, DUET is required to regulate microtubule organization and cell cycle transitions during male meiosis, and functions as a direct transcription activator of the meiotic gene *TDM1*. Expression profiling showed reduced expression of a subset comprising about 12% of a known set of meiosis preferred genes in the *duet* mutant. Our results reveal the action of DUET as a transcriptional regulator during male meiosis in plants, and suggest that transcription of meiotic genes is under stagewise control in plants as in yeast.

## Author Summary

Meiosis is a critical event in sexual reproduction. During meiosis, chromosomes recombine and segregate twice consecutively to produce haploid daughter cells, which differentiate into gametes. In humans, errors in meiosis are the leading causes of congenital birth defects. In plants, bypassing the meiotic program can lead to production of clonal seeds that retain hybrid traits that otherwise segregate. Thus, understanding the controls of meiosis has major implications for both health and crop improvement. How meiotic gene expression is regulated in multicellular eukaryotes to promote entry into and progression through the meiotic program is poorly understood. Here we identify DUET, a protein essential for male meiosis in the model plant *Arabidopsis thaliana*, as a regulator of meiotic gene expression. We found that DUET is required for proper expression of *JAS* and *TDM1*. These genes function in male meiosis, and regulate spindle organization during meiosis II and cell cycle transitions, respectively. Expression of DUET at the end of prophase coincides with the onset of *TDM1* expression, and DUET directly binds *TDM1*, indicating *TDM1* is a direct target of DUET. Our results provide an initial framework for further elucidating the developmental and molecular controls of meiotic gene expression in plants.

## Introduction

Reproductive development in sexual organisms culminates in the production of highly specialized haploid cells, the gametes, which fuse to produce the zygote. An essential event in the production of gametes is meiosis, which is directly responsible for producing haploid cells. Meiosis is a complex process during which homologous chromosomes recombine, synapse, and segregate in two successive rounds without an intervening S-phase. Coordination of meiotic events is essential for successful production of haploid daughter cells.

In yeast, temporal control of meiotic gene expression plays a critical part in coordinating meiotic events with meiotic progression. Entry into meiosis in yeast triggers a transcriptional cascade resulting in sequential expression of meiotic genes. In *S*. *cerevisiae*, entry into meiosis activates Ime1, a transcription factor that is responsible for expression of early genes that are required for premeiotic S-phase, synapsis and recombination. Among ImeI targets, Ndt80 is a transcription factor that activates middle genes, which are required for progression through meiotic divisions and spore formation, followed by late genes which control spore development [1]. A similar transcriptional cascade is triggered upon entry into meiosis in *S*. *pombe*. However, the key transcription factors Ste11 and Mei4 are not homologues of their *S*. *cerevisiae* counterparts ImeI and Ndt80 respectively, indicating poor conservation of these factors even though the transcriptional cascades they control are conserved [2].

In contrast to yeast, few factors controlling meiotic gene expression have been identified in multicellular eukaryotes, and how they contribute to normal meiosis and participate in specifying distinct male and female meiotic programs remain unclear [3–6]. To date in plants, no transcriptional regulator of meiotic gene expression has been identified [7]. In *Arabidopsis*, mutants displaying abnormal expression of meiotic genes in ovules have been recently described. *ACTIN RELATED PROTEIN 6* (*ARP6*) encodes a subunit of the H2A.Z histone variant loading complex SWR1, and *KLUH* (*KLU*) encodes a putative cytochrome P450 monooxygenase [8,9]. Mutations in *ARP6* and *KLU* result in somatic expression of the recombination factor *DMC1*, which is normally restricted to meiocytes, but have a limited impact on *DMC1* expression in female meiocytes. The relationship, if any, between these genes are unknown, and the mechanisms by which they regulate meiotic expression of *DMC1* remain to be fully elucidated. Thus, the controls of meiotic gene expression in plants are largely unknown.

In Arabidopsis, *DUET* (also known as *MALE MEIOCYTE DEATH 1*, *MMD1*) encodes a PHD finger protein essential for male meiosis [10,11]. The loss of *DUET* results in cytoplasmic collapse of meiocytes, defects in chromosome condensation, delay in progression and arrest at metaphase I, absence of an organelle band at interkinesis, and formation of aberrant meiotic products including dyads and triads, which do not survive. The relationship between these phenotypes, and the function of *DUET* during meiosis are unknown. However, *DUET* was shown to be expressed during male but not female meiosis, suggesting a sex-specific function [10,11]. Here, we report that DUET is required for meiotic gene expression, and interacts with chromatin via its PHD finger. We found that DUET PHD finger binds H3K4me2 in vitro, and we show that this interaction is required for function. We obtained partially complemented *duet* lines, with a PHD finger that had reduced binding affinity for H3K4me2. We found that meiosis I defects were rescued, but that meiosis II still produced aberrant meiotic products, with defects in chromosome segregation and cytokinesis. Analysis of microtubule organization in *duet* and *duet* partially complemented lines, revealed a parallel spindle phenotype during meiosis II consistent with formation of dyads and triads in *duet*. The parallel spindle phenotype in *duet* correlated with the loss of expression of *JAS*, a gene required for meiosis II spindle organization in male meiosis [12]. Further analysis revealed that expression of *TDM1*, a gene required for meiotic cell cycle transitions [13], was also lost in *duet*. Remarkably, we found that *DUET* expression was restricted to the diplotene stage of prophase I. We further show by chromatin immunoprecipitation experiments (ChIP) that DUET binds *TDM1* promoter, indicating that *TDM1* is a direct target of DUET. Consistent with this result, we found that the onset of *TDM1* expression coincided with the timing of DUET expression. Moreover, *TDM1* was not expressed during female meiosis, indicating a possible role of DUET in regulating male meiotic gene expression.

Our results reveal that DUET functions as a transcriptional regulator during male meiosis in *Arabidopsis*, and that binding of its PHD finger to H3K4me2 is critical for function. DUET expression was detected only at diplotene, and the functions of the targets we identify in meiosis I and II processes, are reminiscent of the control of middle meiotic gene expression by transcription factors in yeast. Our results also suggest a role for histone modifications in the regulation of meiotic gene expression in plants.

## Results

### *DUET* encodes an H3K4me2 chromatin reader

**Fig 1:**
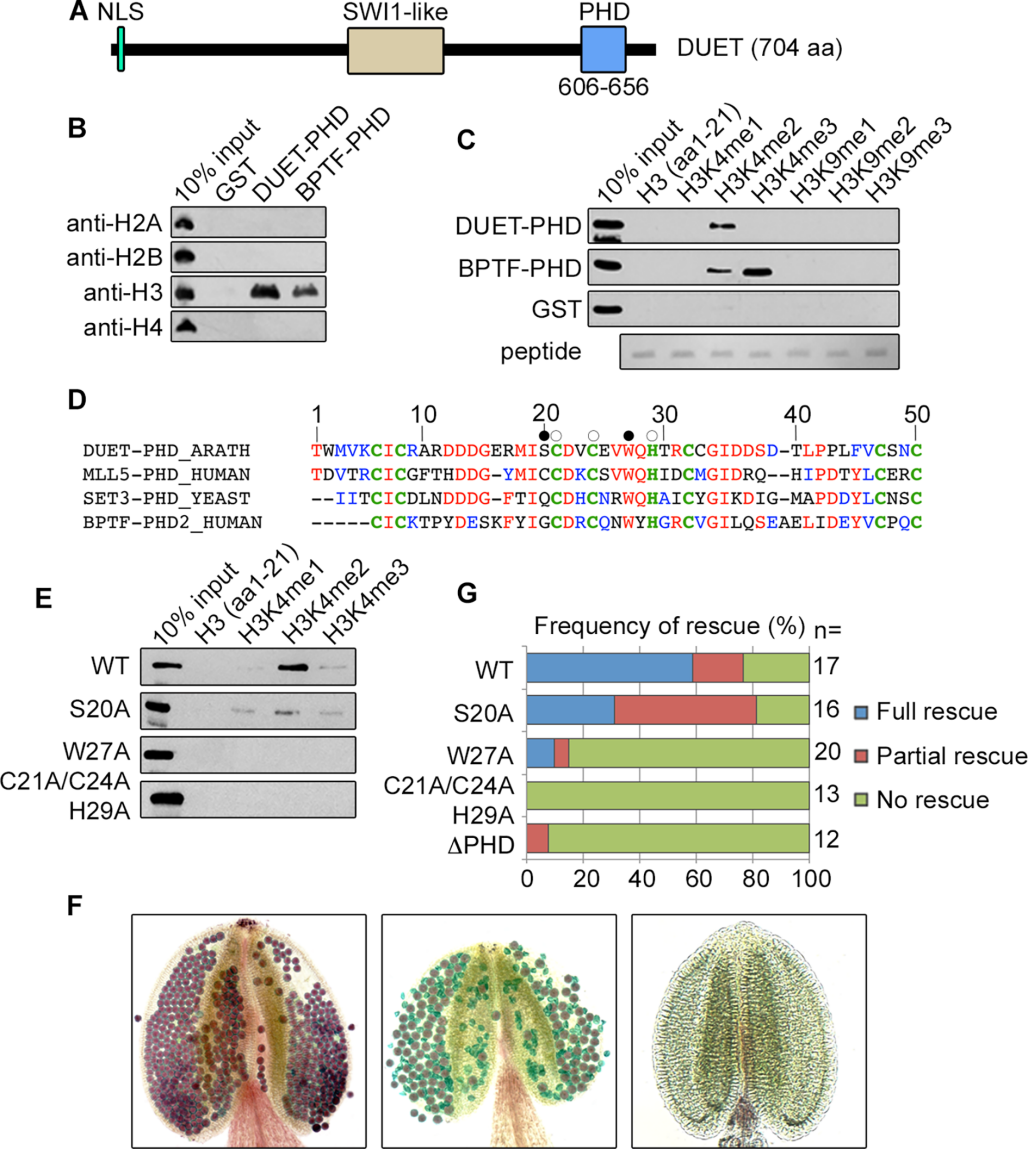
DUET PHD is a reader of H3K4me2. (A) Schematic representation of DUET protein. *DUET* encodes a 704 amino acid plant specific protein with a nuclear localization sequence (NLS), a region showing homology with the meiotic gene *SWITCH1* (*SWI1*), and a C-terminal Plant Homeo Domain (PHD). (B) Calf thymus histone pull down followed by western blot with the indicated antibodies. BPTF PHD2 is used as positive control. (C) Peptide pull downs followed by western blot with anti-GST antibody. (D) The PHD finger of DUET is conserved across eukaryotes. Sequence alignment of DUET PHD finger with characterized homologues showing highest homology obtained by PSI-BLAST. DUET PHD shares 46% identity with human MLL5 PHD (E = 9e-08), 44% identity with *S*.*cerevisiae* Set3 PHD (E = 2e-07), and 41% identity with human BPTF PHD2 (E = 3e-05). Conserved and similar residues are in red and blue respectively, conserved C4HC3 structural residues are in bold green. White circles indicate mutation sites in the triple mutant (C21A/C24A/H29A), and black circles indicate the point mutations S20A and W27A. (E) Histone peptide pull downs with methylated H3K4 peptides and the indicated mutant DUET PHD fingers. (F) Representative Alexander staining of T1 *duet* plants transformed with a wild-type *DUET* construct (WT). Left panel: fully viable pollen (purple) indicating full rescue; middle panel: mix of viable and dead pollen (green) indicating partial rescue; right panel: absence of pollen indicating no rescue. (G) Quantification of rescue phenotypes obtained for independent T1 *duet* plants transformed with the indicated construct. WT, WT full length *DUET* construct; n, number of independent transformants analyzed per construct.

The *Arabidopsis* gene *DUET*/*MMD1* is essential for male meiosis and encodes a plant specific protein with a Plant Homeo Domain (PHD) finger from amino acid 606 to 656 (Fig 1A) [10,11]. PHD fingers are chromatin reader domains that bind modified or unmodified C-terminal tails of histone H3 or H4 [14]. Pull-down assay of DUET-PHD finger with calf thymus histones revealed that DUET PHD exclusively recognized histone H3 (Fig 1B). Pull-down assays using histone H3 peptides carrying specific modifications revealed that DUET PHD bound H3K4me2, and not other H3 modified or unmodified peptides (Fig 1C and S1 Fig). BLAST and phylogenetic analysis of DUET PHD revealed relatedness with yeast Set3 PHD finger, which also binds H3K4me2, as well as with H3K4me2/3 binding human Mixed Lineage-Leukemia 5 (MLL5) and Bromodomain and PHD Transcription Factor (BPTF) PHD fingers (Fig 1D and S1 Fig) [15–18]. Alignment of DUET PHD with Set3, MLL5, and BPTF PHD fingers, showed that residues required for methylated H3K4 binding are conserved, including a tryptophan at position 27 which forms an aromatic cage that is involved in binding H3K4me3 in BPTF [19], pointing to a similar mechanism for H3K4me2 recognition.

To determine the relevance of H3K4me2 binding for DUET function, we generated a mutant DUET PHD in a conserved cage residue (W27A), a mutant in a residue outside the aromatic cage (S20A), and a triple mutant of structural residues predicted to be required for folding (C21,C24 and H29) (Fig 1D), and tested the effect of these mutations on methylated H3K4 peptide binding. The W27A and the structural triple mutants lost the ability to bind H3K4me2, whereas the S20A mutation reduced H3K4me2 binding (Fig 1E). We then introduced these mutations as well as a deletion removing the PHD finger (ΔPHD) in a complementing construct containing the full length *DUET* coding sequence driven by its endogenous promoter. In *duet*, meiotic defects lead to complete absence of pollen [10,11]. The extent of complementation was determined in independent primary *duet* transformants by Alexander staining, a method that stains viable pollen purple and dead pollen green [20]. Out of 17 T1 *duet* plants carrying a WT DUET construct, 10 plants were fully complemented, 4 plants showed absence of complementation, and 3 plants showed an intermediate phenotype characterized by the presence of both dead and viable pollen, which we interpreted as partial complementation (Fig 1F and 1G). In comparison, the structural triple mutant, the ΔPHD, and the W27A constructs largely failed to complement (Fig 1G), and the S20A construct showed reduced complementation, frequently resulting in partial complementation (50%, n = 16) when compared to the WT construct (18%, n = 17) (Fig 1F and 1G). Overall, the complementation assay recapitulated the in-vitro peptide binding results, strongly indicating that recognition of H3K4me2 during meiosis is critical for DUET function.

### DUET is required for meiosis II independently of meiosis I

**Fig 2:**
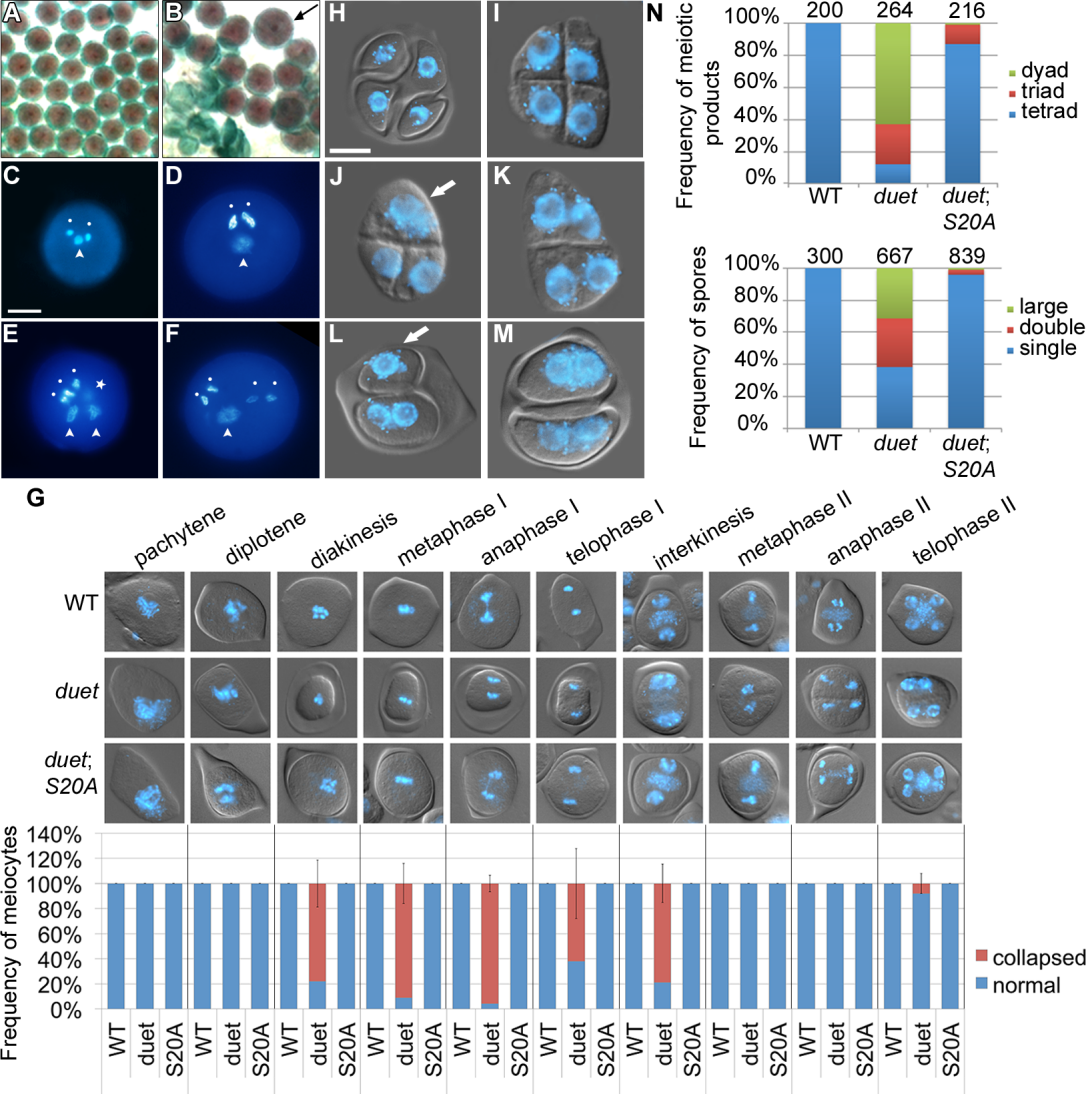
Partial complementation of *duet* reveals a role in chromosome segregation and cytokinesis. (A and B) Alexander staining of mature anthers of WT (A) and partially complemented *duet* with *DUET-S20A* (*duet*;*S20A*) (B). Viable pollen is purple; dead pollen is green; arrow points to an enlarged pollen grain. (C) DAPI stained WT pollen with a male germ unit (MGU) comprising two condensed sperm nuclei (white dots) and one decondensed vegetative cell nucleus (arrowhead).(D-F) DAPI stained enlarged pollen from *duet*;*S20A*; scale bar = 10 μm. (D) DAPI stained large *duet*;*S20A* pollen grain with a normal MGU. (E) DAPI stained large *duet*;*S20A* pollen grain with two MGUs; star indicates a condensed sperm-like cell out of focus. (F) DAPI stained large *duet*;*S20A* pollen grain with four sperm-like condensed cells, and one decondensed vegetative-like cell. (G) Analysis of collapse during meiosis in WT, *duet* and *duet*;*S20A*. Top panel: analysis of cytoplasmic collapse during meiosis in the indicated backgrounds. Pictures are merged DIC and DAPI, which stains chromosomes blue. Bottom graph: stage-wise quantification of collapsed meiocytes. Data are presented as mean ± SD for at least 20 meiocytes per stage. (H-M) Microscopic analysis of tetrads from WT (H), *duet* (I-K) and *duet*;*S20A* (L and M). scale bar = 10 μm.

The main phenotypes of *duet* include cytoplasmic collapse of meiocytes, delayed progression, formation of dyads and triads, and absence of pollen [10,11]. Absence of an allelic series in *DUET* prevents analysis of the relationships between these phenotypes. However, we found that a complementing construct carrying the S20A mutation in DUET PHD finger frequently resulted in partial complementation (Fig 1F and 1G). Analysis of mature pollen in *duet*;*S20A* lines revealed a low frequency of enlarged grains (about 5%) in addition to dead pollen (Fig 2A and 2B). DAPI staining of *duet*;*S20A* pollen further revealed that enlarged pollen grains often contained more than one vegetative cell and two sperm cells (Fig 2C–2F). We examined meiosis for defects that could account for these phenotypes. Interestingly, the cytoplasmic collapse of meiocytes during meiosis I in *duet* was completely rescued in *duet*;*S20A* (Fig 2G). Completion of meiosis in WT results in tetrads of four spores (Fig 2H). In *duet*, meiosis produced high frequencies of dyads and triads with an equal number of spores and nuclei, i.e. 2 and 3 of each respectively, suggesting chromosome segregation and/or cell cycle defects (Fig 2I and 2J). In addition, we observed meiotic products with binucleated spores, indicating cytokinesis defects (Fig 2K). We observed similar phenotypes in *duet*;*S20A* but at lower frequencies (Fig 2L–2N). Subsequent development of enlarged and binucleated microspores likely accounts for enlarged pollen and pollen with more than 3 cells, respectively. These observations indicate that cytokinesis, and chromosome segregation and/or cell cycle regulation, are compromised during meiosis II in both *duet* and *duet*;*S20A*. Because we did not detect any phenotype during meiosis I in *duet*;*S20A*, we conclude that DUET is required for meiosis II independently of its function in controlling progression through meiosis I.

(H) WT tetrad with four spores containing each a single haploid nucleus. (I) *duet* tetrad. (J) *duet* triad of spores with two small and one large nucleus. (K) *duet* dyad of spores containing two small nuclei each. (L) dyad of spores containing one large (white arrow) and two small nuclei, respectively, in *duet*;*S20A*. (M) dyad of spores containing two small nuclei each, in *duet*;*S20A*. Compare with (K). (N) Quantification of the tetrad phenotypes described above. Top graph: frequency of spore numbers per tetrad irrespective of number of nuclei. Tetrad = three spores, triad = three spores, dyad = two spores. Bottom graph: frequency of nuclei per spore, irrespective of spore number. Single = similar to WT; double = two nuclei per spore; large = large nucleus, arrow in (J) and (L). Numbers above columns indicate the number of analyzed tetrads (top graph), and spores (bottom graph) respectively.

### DUET is required for spindle and radial microtubule organization during meiosis II

**Fig 3:**
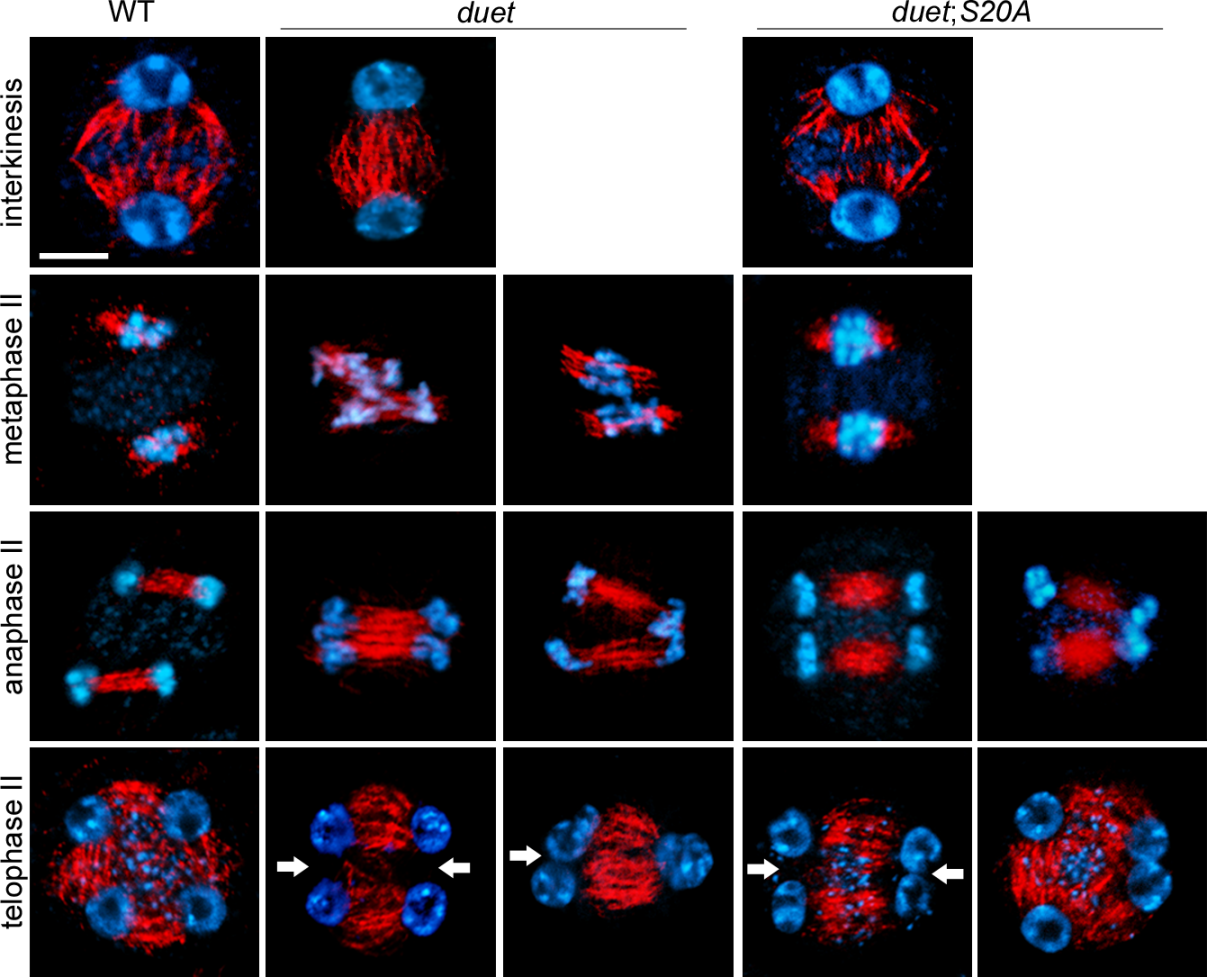
DUET is required for proper spindle organization during meiosis II. Immunostaining of α-tubulin on male meiotic squashes from WT, *duet*, and *duet*;*S20A*. The meiotic stages are indicated for each row. Chromosomes were stained with DAPI (blue) tubulin, red. White arrow: absence of radial microtubule arrays (RMA) between nuclei; scale bar = 10 μm. At least 20 meiocytes were analyzed per stage and per genotype.

We took advantage of a partially complemented *duet*;*S20A* line to explore the function of *DUET* during meiosis II. In *Arabidopsis*, mutants affecting spindle organization in meiosis II have been shown to produce dyads and triads [12,21]. To test whether dyads and triads in *duet* and *duet*;*S20A* result from spindle defects in meiosis II, we analyzed microtubule organization by immunostaining of male meiotic squashes. At interkinesis, microtubules form arrays emanating from the nuclei and interrupted by the organelle band (Fig 3). Microtubule organization at interkinesis was similar to WT in *duet* and *duet*;*S20A*, except that the organelle band does not form in *duet*. In WT metaphase II, the spindle forms around the two chromosome complements resulting from segregation during meiosis I, which are clearly separated by the organelle band (Fig 3). However, in *duet* metaphase II the chromosomes often appeared close and not well separated. This was further accentuated in anaphase II, during which fused and tripolar spindles segregated chromatids as 2 or 3 groups respectively, instead of 4 in WT. This indicates that chromatid segregation defects in meiosis II are likely to result from compromised spindle organization.

After chromatids segregate in anaphase II, microtubules assemble as radial microtubule arrays (RMA) between adjacent nuclei (Fig 3). In *Arabidopsis* male meiosis, cytokinesis occurs after telophase II and the plane of division is perpendicular to RMAs. In *duet* and *duet*;*S20A* however, we often observed absence of RMA between nuclei. Thus, cytokinesis defects are likely to arise from defective RMA establishment in *duet* and *duet*;*S20A*. Overall, these results establish that DUET regulates chromatid segregation and cytokinesis through microtubule organization during meiosis II.

### DUET localizes on chromosomes at diplotene

**Fig 4:**
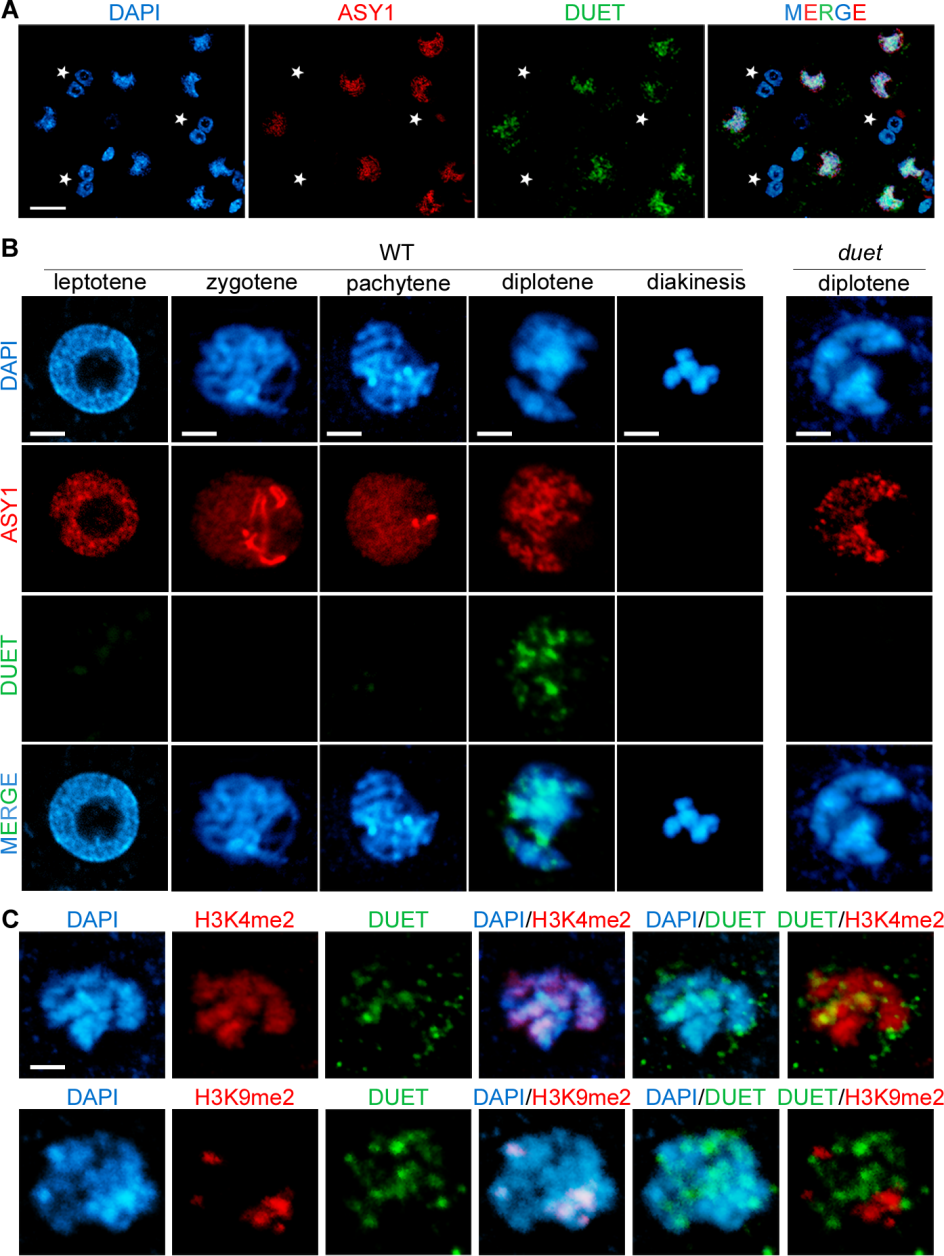
DUET is expressed during diplotene and localizes on euchromatin. (A and B) Dual immunostaining of DUET (green) and ASY1 (red). (A) DUET is only expressed in ASY1 positive cells (white arrows), and not in other anther cells, including multinucleated tapetal cells (stars). Scale bar = 20 μm. (B) Detailed analysis of DUET expression during prophase stages, according to chromosome morphology and ASY1 pattern. Scale bar = 5 μm. (C) Dual immunostaining of DUET (green), and H3K4me2 (red, top panel), or H3K9me2 (red, bottom panel) chromatin marks. In each row, the first three pictures show single channels, the last three pictures are merged images of two channels. Scale bar = 5 μm.

We found that DUET is required for cellular organization during meiosis I, and for microtubule organization during meiosis II. To further characterize the function of DUET, we determined its expression pattern. We raised rabbit and rat polyclonal antibodies against recombinant unique regions of DUET, and performed immunostaining on male meiotic squashes. Using rabbit polyclonal antibodies, DUET staining was detected only in nuclei of meiotic cells marked by ASY1, a component of the synaptonemal complex associated with axial elements [22], and not in somatic cells including tapetal cells (Fig 4A, n>100). We then analyzed DUET expression during prophase in detail. At leptotene, chromosomes formed thin threads that were decorated by ASY1. At zygotene, chromosomes started to synapse, and ASY1 signal was diffuse on synpapsed regions. At pachytene, synapsis was complete and chromosomes appeared as thick strands with an overall diffuse ASY1 signal. During diplotene, chromosomes desynapsed and ASY1 signal remained on chromosomes as discontinuous patches along remnants of chromosome axis. At diakinesis, chromosomes condensed before aligning for metaphase I, and ASY1 signal was undetectable. Strikingly, we detected DUET signal only during the diplotene stage (Fig 4B, n = 96), but not during pachytene or diakinesis, (n = 250 and 50 respectively). We also never observed DUET staining at any stage of meiosis after prophase I, indicating that DUET is expressed only during diplotene, or that DUET expression at other stages is too low to be detected (S2 Fig). We could not detect any signal in *duet* meiocytes, indicating the antibody is specific (Fig 4B, n = 100). Immunostaining of male meiotic squashes with a rat anti-DUET antiserum further confirmed DUET is expressed at the diplotene stage of meiosis (S2 Fig).

The pattern of DUET overlapped with the DNA dye DAPI, indicating that DUET localizes on chromatin (Fig 4B). We showed above that DUET-PHD binds H3K4me2 and that this interaction is critical for function. Consistent with these results, we found that DUET signal overlapped with H3K4me2 (n = 8, Fig 4C upper panel), but not with pericentromeric H3K9me2 (n = 10, Fig 4C lower panel). Thus, DUET localizes to euchromatin and its peak of expression at diplotene suggests that its role in meiosis I and II is likely to be indirect.

### DUET is required for expression of the meiotic genes *TDM1* and *JAS*

**Fig 5:**
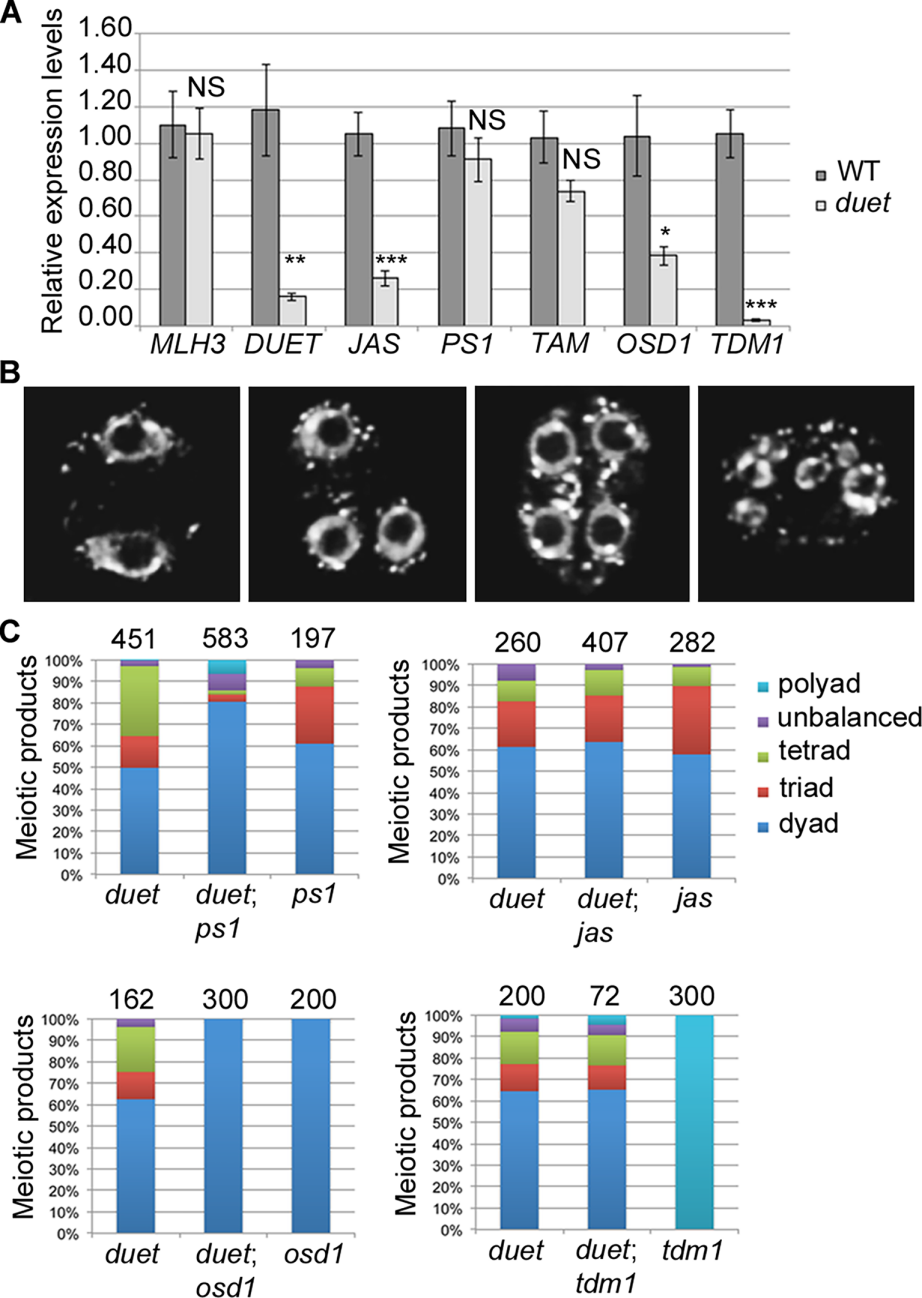
DUET is required for proper expression of *JAS* and *TDM1* during meiosis. (A) Quantitative RT-PCR (qPCR) analysis of meiotic gene expression in WT and *duet* from anthers dissected from 0.5–0.6mm buds normalized on *ACT11* expression. Data are presented as the mean ± SEM (error bars) of at least two independent experiments with at least three samples each. Statistically significant differences: P < 0.05 = (*); P < 0.01 = (**); P < 0.001 = (***); ns = no significant difference (t-test). (B) Representative images of major classes of male meiotic products obtained in F2 double mutant combinations. Chromosomes were stained with DAPI (white). Left to right: meiotic products with two, three, four and more than four nuclei respectively. (C) Quantification of male meiotic products described in (B), in the indicated genetic backgrounds.

To test whether DUET affects gene expression, as other chromatin readers do [17], we performed quantitative RT-PCR on meiotic anther cDNA from WT and *duet*. We generated cDNAs from anthers dissected from 0.5–0.6 mm WT and *duet* buds, which included prophase, as well as meiosis I and meiosis II stages (S3 Fig). In *Arabidopsis*, mutants in *JASON* (*JAS*) and *PARALLEL SPINDLE 1* (*PS1*), affect meiosis II spindle organization and produce high frequencies of dyads and triads as a consequence [12,21]. Based on phenotypic similarities with *duet*, we examined *JAS* and *PS1* expression. We found that expression of *PS1* and *MUT-L HOMOLOGUE 3* (*MLH3*), a meiotic specific gene required for completion of recombination [23], was similar in WT and *duet*. However, expression of *JAS* was reduced by about 80% in *duet* (Fig 5A). The loss of *JAS*, but not *PS1*, expression suggests a specific requirement of *DUET* for *JAS* expression, rather than a consequence of altered meiotic progression. While this finding provides a reasonable explanation for the spindle defects observed in *duet*, it cannot account for all *duet* phenotypes. Therefore, we surveyed expression of other known genes acting during MI and MII. Particularly, progression through MI and MII is controlled by complex genetic interactions between *TARDY ASYNCHRONOUS MEIOSIS* (*TAM*), *OMISSION OF SECOND DIVISION 1* (*OSD1*), and *THREE DIVISION MUTANT 1* (*TDM1*). *OSD1* encodes a UVI-like protein which functions as a putative APC/C inhibitor [13]. Mutants in *OSD1* skip meiosis II and produce dyads of diploid spores [24,25]. *TAM* encodes a cyclin A required for proper meiotic cell cycle progression [26]. Similarly to *OSD1*, mutants in *TAM* skip meiosis II and produce dyads, as well as triads occasionally, indicating cell cycle defects can also result in triads in *Arabidopsis* [27]. *TDM1* encodes a tetratricopeptide repeat protein required for cell cycle exit after meiosis II [28]. In *TDM1* mutants, meiocytes undergo a third round of division without intervening S phase, and the attempt to segregate haploid chromatids results in polyads. When we examined expression of *OSD1*, *TAM* and *TDM1* in *duet*, we found that expression of *TDM1* was severely reduced, whereas *TAM* expression was similar to WT, and *OSD1* expression was mildly reduced (Fig 5A).

The fact that the expression of only a subset of meiotic genes is affected in *duet* strongly suggests that reduced expression in the mutant does not result from altered meiotic progression. To confirm this, we performed an epistasis analysis by analyzing the outcome of meiosis in double mutant combinations. We reasoned that, if a gene shows reduced expression as a consequence of altered meiotic progression in *duet*, then the corresponding double mutant should be significantly different from the single *duet* mutant. However, if a gene shows reduced expression because its expression in meiosis requires *DUET*, then the double mutant should be indistinguishable from the single *duet* mutant. We generated *duet*;*ps1*, *duet*;*jas*, *duet*;*osd1* and *duet*;*tdm1* double mutants. All double mutant combinations were male sterile, and exhibited similar meiosis I defects as in *duet*, including cytoplasmic collapse, defects in chromosome condensation, and delayed progression through meiosis I (S4 Fig). We then analyzed the frequency of meiotic products in F2 double homozygous plants, based on the number of nuclei per meiotic product (Fig 5B). Because *duet* and *osd1* on one hand, and *ps1*, *jas* and *tdm1* on the other hand, are in different ecotypes (Ler and Col respectively), we used single mutants from the segregating population as controls for each double mutant combination. Each single mutant produced dyads, triads, tetrads and polyads, similarly to their parent (Fig 5C). The loss of *ps1* in *duet* resulted in higher frequency of dyads, which is consistent with our result that *PS1* is still expressed in *duet* (Fig 5C). In contrast, the *duet*;*jas* double mutant produced dyads, triads and tetrads in the same proportions as *duet* (Fig 5C), indicating that the loss of *JAS* function in *duet* has no effect, which is consistent with *JAS* expression being compromised in *duet*. Our qPCR data indicated that *TDM1* expression was lost in *duet*, and the *duet*;*tdm1* double mutant produced dyads, triads and tetrads similarly to *duet*, whereas the *tdm1* single mutant exclusively produced polyads (Fig 5C and S4 Fig). However, while our qPCR data indicated that *OSD1* expression was reduced in *duet*, the *duet*;*osd1* double mutant only produced dyads (Fig 5C). Skipping meiosis II did not rescue *duet* sterility defects, as the *duet*;*osd1* double mutant exited meiosis after meiosis I and was male sterile (Fig 5D and S4 Fig). Overall, our genetic analysis is consistent with expression of *JAS* and *TDM1* being lost in *duet* meiosis.

### Loss of *DUET* particularly affects genes showing meiotic preferred expression

To further identify targets of DUET with a functionally relevant role in meiosis, we compared transcriptomes of 0.5–0.6 mm meiotic stage buds from WT and *duet* (S3 Fig) by microarray analysis. The microarray experiments revealed that, 1370 genes were downregulated and 716 genes were upregulated at least twofold in *duet*, out of 29274 genes represented on the array (S1 Table). The genes showing altered regulation would be expected to include meiotic genes, as well as genes that function downstream of meiosis for which the developmental stages are missing in *duet*. Indeed, among the genes showing downregulation in *duet* were *TDM1*, as well as *MS1* and *MS2* which are required for development of microspores into pollen [29,30]. To target genes involved in meiosis, the deregulated genes in *duet* were compared with a list of 296 genes preferentially expressed in meiosis [31], of which 290 were present on our array. This resulted in identification of 20 up and 34 down regulated genes in *duet* compared with WT. There is thus significant enrichment for meiotically preferred genes among downregulated genes (p(Χ^2^) < 0.001). We used meiotic anther cDNAs to validate our microarray results by qPCR on a subset of downregulated individual genes, and all the genes we tested showed downregulation (n = 6, S1 Table), indicating that DUET is likely to function as a positive regulator of gene expression. To understand which meiotic processes DUET is required for, we searched for gene ontology (GO) term enrichment among the 34 downregulated genes, however the analysis did not return any significant enrichment of GO terms. The genes we identified as potential targets of DUET have not been functionally characterized for a role in meiosis. Interestingly, one of the downregulated genes is *BRP4*, whose function was shown to be required for pollen development, but its expression starts during late meiosis [32]. These results are consistent with our functional analysis, and with the view that DUET is likely to function as a regulator of genes that are expressed in middle or late meiosis.

### DUET is directly required to activate *TDM1* expression

**Fig 6:**
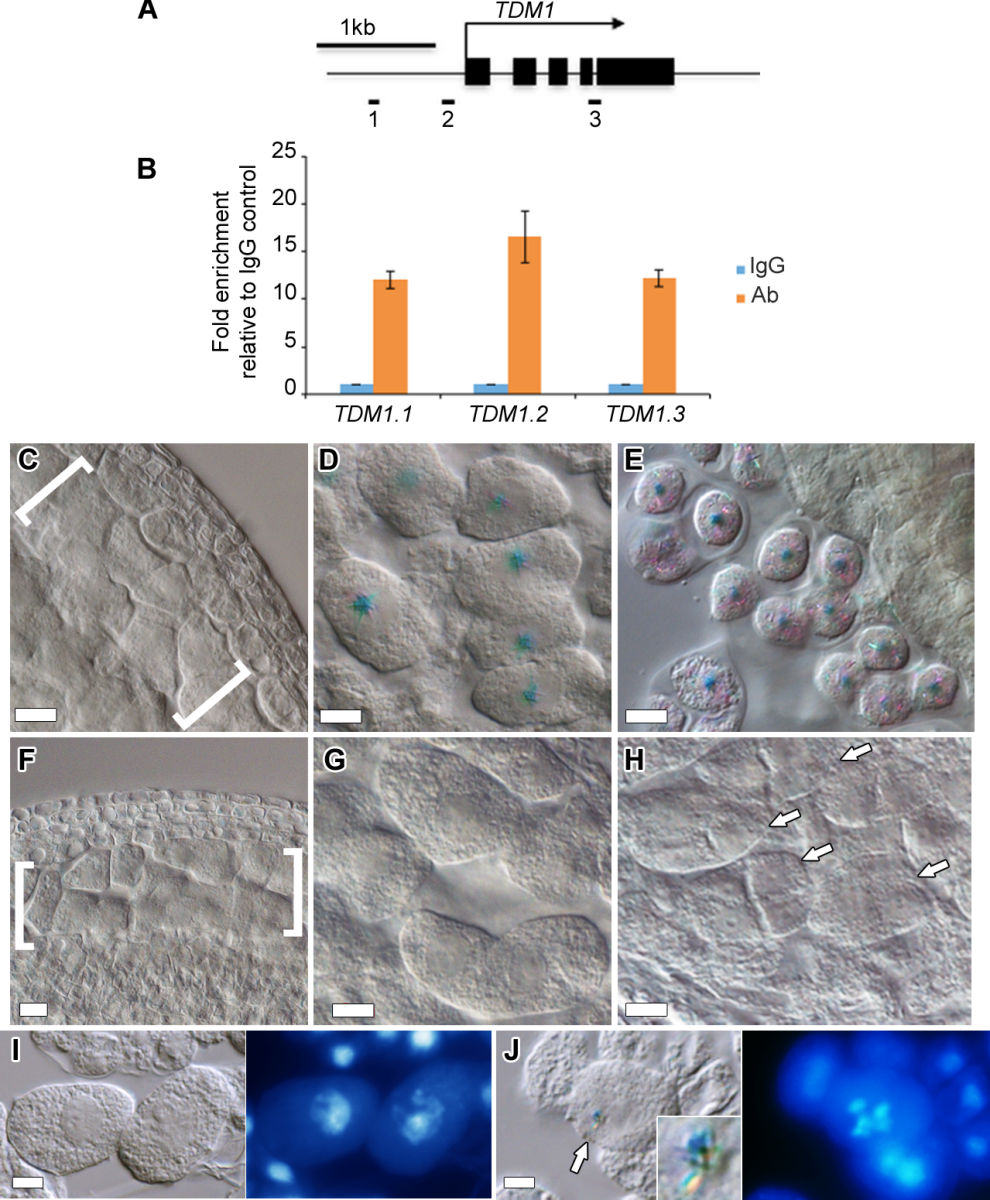
*TDM1* is a direct target of DUET. (A) Schematic diagram of *TDM1*, representing the regions analyzed by ChIP-qPCR (black rectangles). Black boxes represent exons, the arrow represent the direction of transcription. (B) Quantification of ChIP relative to the IgG control by qPCR. Columns represent the mean, and error bars represent the standard error of the mean from 3 independent biological samples. (C-E) Expression of *pTDM1*::*NLS-GUS* during WT meiosis in whole mount anthers. (C) Early prophase, (D) late prophase, meiocytes are individualized by a callose envelope, (E) tetrad stage. (F-H) Expression of *pTDM1*::*NLS-GUS* during *duet* meiosis in whole mount anthers. (F) Early prophase, (G) late prophase, (H) tetrads. Brackets in (C) and (F) delineate meiocytes are arrows in (H) point to individual tetrads. All panels, scale bars 10 μm, except (D) and (G), 5 μm. (I,J) GUS staining (left panel), followed by DAPI staining of squashed anthers (right panel), allows staging of meiocytes based on chromosome morphology. (I) pachytene, (J) diakinesis. The arrow in (J) points to the GUS signal shown in the inset.

Our results indicate that DUET is required for expression of *TDM1* and *JAS* during meiosis. To test whether *TDM1* and *JAS* are direct targets of DUET, we performed chromatin immunoprecipitation (ChIP) from WT inflorescences using DUET antiserum. We failed to detect enrichment of DUET at *JAS* relative to a control locus (*At4g03870*), but found that DUET showed a 15-fold enrichment at *TDM1* relative to the same control locus. We selected three regions for a more detailled analysis of DUET binding to *TDM1* (Fig 6A). We found that DUET enrichment peaked at a specific region of *TDM1* that is marked by H3K4me2 in *Arabidopsis* seedlings (Fig 6B) [33,34]. This result is consistent with our findings described above that DUET PHD binds specifically to H3K4me2. These results suggest that *TDM1* is likely to be a direct target of DUET. To determine if DUET controls *TDM1* expression by binding to its promoter, we established the expression patterns of *TDM1* promoter using a *GFP-GUS* reporter, tagged with a nuclear localizing sequence (*NLS-GUS* for simplicity). We detected *pTDM1*::*NLS-GUS* expression in anther meiocytes (Fig 6C–6E), as reported previously for the *TDM1* transcript [35]. We first observed GUS staining in meiocytes in late prophase, and until the tetrad stage (Fig 6C–6E). However, we failed to detect *pTDM1*::*NLS-GUS* expression during meiosis in *duet* (Fig 6F–6H), indicating that DUET binding to *TDM1* promoter is necessary for *TDM1* expression during male meiosis. Detailed microscopic analysis revealed that the onset of *pTDM1*::*NLS-GUS* expression is at late diplotene, which coincides with the timing of DUET expression (Fig 6I and 6J).

Interestingly, the loss of *TDM1*, like the loss *DUET*, results in male sterility indicating an important role for *TDM1* in male meiosis [13,28,36]. While *DUET* has been shown to be expressed only in male meiosis [10,11], the expression pattern of *TDM1* is unknown in female meiosis. To address this, we analyzed *pTDM1*::*NLS-GUS* expression in female meiosis. In contrast to male meiosis, we could not detect any signal from prophase to tetrad stages in meiotic ovules (S5 Fig). These data indicate that *TDM1* expression in male meiosis depends on direct interaction of DUET on its promoter.

### Identification of DUET expressologs in plants

Putative DUET homologues exist in a large number of plant species, including both dicots and monocots. Analysis of the expression pattern of these homologues from microarray data available for these systems further revealed the existence of putative DUET expressologs, i.e. proteins with high homology and similar expression patterns (Table 1 and S6 Fig). Thus, we identify putative DUET expressologs in Poplar, Soybean, Potato, Tomato, Maize and Rice, suggesting that the function of DUET in reproductive development is conserved in plants, including crops of major agronomical importance.

**Table 1:** Identification of DUET expressologs.

Protein	Identity (%)	Expression pattern
*Arabidopsis*_AT1G66170	n.a.	Closed buds, mature pollen
Poplar_ 0017s02450	50.3	Male and female catkins
Soybean_Glyma02g41020	50.1	Flowers
Rice_ Os03g50780	32.5	Young inflorescence, SAM
Maize_GRMZM2G408897	32.8	SAM, shoot internode, tassel, anthers, pre-pollination cob, embryo, endosperm, pericarp
Potato_PGSC0003DMP400023432	48.2	Flowers, stamen, fruit, tuber, leaves, SAM, stem
Tomato_Solyc11g011560	48.2	Closed buds

A search for DUET expressologs (identical proteins with similar expression pattern) in Poplar, Medicago, Soybean, Rice, Barley, Maize, Potato and Tomato returned the proteins listed above (http://bar.utoronto.ca/expressolog_treeviewer/cgi-bin/expressolog_treeviewer.cgi). Expression of these proteins in reproductive organs indicates that DUET function could be conserved in plants, including in major crops. n.a., not applicable: SAM, shoot apical meristem.

## Discussion

We have shown that DUET is required for proper gene expression during male meiosis in *Arabidopsis*. We further identify two genes with critical meiotic functions, *JAS* and *TDM1*, which depend on DUET for expression, and further show that *TDM1* is a direct target of DUET. DUET expression peaks at diplotene, and this coincides with the onset of TDM1 expression. Our results thus provide insight into the temporal regulation of meiotic gene expression in plants. Furthermore, our characterization of DUET PHD finger, and the fact that it binds H3K4me2, point to a possible role of histone modifications in the control of meiotic gene expression. Overall, our work reveals that DUET functions as a transcriptional regulator during male meiosis in plants, and provides an initial framework for further elucidating the molecular mechanisms controlling meiotic gene transcription in plants.

### DUET is required for expression of the meiotic genes *JAS* and *TDM1*

How gene expression is controlled to promote entry into meiosis, and how regulation of meiotic gene expression participates in the coordination of meiotic processes, is poorly understood in multicellular eukaryotes [7,37]. In mouse and *Drosophila*, transcription factors of the MYB family have been shown to be required for meiotic gene expression [3,5]. However, mutants in those factors display strong meiotic arrest phenotypes as a result of meiotic checkpoint activation, and the exact contribution of these transcription factors to normal meiosis is therefore difficult to determine [3]. We took advantage of the apparent absence of meiotic checkpoints in *Arabidopsis* meiosis to carry out a detailed analysis of the function of DUET, a PHD finger protein essential for male meiosis. We found that the loss of DUET resulted in deregulated expression of a subset of genes with high meiotic expression. We further obtained molecular and genetic evidence that expression of *TDM1* and *JAS*, two genes with critical meiotic functions in meiotic cell cycle transitions and spindle organization respectively [12,28,38], is lost in *duet*.

*TDM1* encodes a tetratricopeptide repeat protein that plays a critical role in meiotic cell cycle transitions [13,28,36,38]. In addition to molecular and genetic evidence for the loss of *TDM1* expression in *duet*, we failed to detect *TDM1* expression during meiosis in *duet* using a promoter reporter indicating that control is at the level of transcription. Furthermore, we found that DUET binds the *TDM1* promoter in ChIP experiments, indicating that *TDM1* is a direct target of DUET. The precise impact of the loss of *TDM1* expression in *duet* is as yet unclear, but these data reveal a critical role for DUET in the control of the meiotic cell cycle. In *TDM1* mutants, male meiocytes do not undergo cytokinesis after MII, and instead enter a third round of chromosome segregation resulting in polyads [28]. In contrast, male meiocytes in *duet* undergo cytokinesis after MII. A likely explanation for the absence of a third division in *duet*, is that the loss of expression of other genes result in epistasy over the loss of *TDM1* expression. In agreement with this view, we found that expression of *OSD1* was reduced in *duet*. Mutants in *OSD1* arrest after MI, and are epistatic over *tdm1* mutants [13]. However, genetic analysis of a *duet*;*osd1* double mutant revealed that *OSD1* expression is not completely lost in *duet*, which in turn is consistent with the fact that meiosis in *duet* proceeds to MII. Perhaps OSD1 levels in *duet* are low enough to prevent entry in a third meiotic division, but high enough to not result in complete MI arrest. Alternatively, the loss of expression of other as yet unidentified meiotic cell cycle regulator(s) besides *OSD1* might result in epistasy over the loss of *TDM1* expression in *duet*.

The loss of expression of *JAS*, which is required for spindle organization in meiosis II [12], provides a mechanism for the formation of dyads and triads in *duet*. Consistent with this, we observed spindle defects similar to *jas* mutants during meiosis II in *duet* and *duet*:*S20A*. These results further suggest that the pleiotropic meiotic phenotype of *duet* results from the loss of expression of several genes. Indeed, DUET is likely to control expression of several additional genes, since the cause of many phenotypes in *duet*, including cytoplasmic shrinkage, partial cytokinesis and elimination of meiotic products, cannot be explained by the loss of *JAS* and *TDM1* alone. In support of this view, we identified several genes with meiotic preferred expression, whose levels of expression were affected by the loss of *DUET*. It is possible that some of the phenotypes in *duet* might result from the simultaneous loss of expression of several genes. Overall, our results are consistent with the view that DUET is likely to function as an activator of gene expression during male meiosis.

### Insights into the temporal controls of meiotic gene expression in *Arabidopsis*

The phenotypes resulting from the loss of *DUET*, and the functions of the target genes *JAS* and *TDM1* that depend on DUET for expression, define DUET’s function in meiotic processes post-prophase during meiosis I, meiosis II and cytokinesis. We also found that the onset of expression of *TDM1*, which we show to be a direct target of DUET, is at late diplotene/diakinesis. Similarly, expression of *BRP4*, a DUET target gene identified by microarray analysis, was shown to be in late meiosis [32]. In *S*. *cerevisiae* and *S*. *pombe*, meiotic genes performing key functions during meiosis I division, meiosis II, and meiotic exit are called “middle genes”, and constitute a group of co-expressed genes under the control of the transcription factors Ndt80 and Mei4 respectively [39,40]. The activity of Ndt80 and Mei4 is controlled at the RNA and protein level to peak after recombination is complete [1,41,42]. Likewise, we found that expression of DUET was highly stage specific, and was detected only at the diplotene stage at the end of prophase. This further suggests that active mechanisms control DUET expression and/or activity. The existence of such mechanisms in plants reveal that, despite the apparent absence of meiotic checkpoints, controls do exist to ensure proper progression of meiotic events through temporal regulation of gene expression. Thus, further elucidation of the regulatory factors acting upstream DUET might shed light on the poorly understood mechanisms regulating meiotic gene expression in plants.

### Histone modifications and meiotic gene expression

The essential requirement of the PHD finger of DUET for its function, suggests that recognition of H3K4me2 plays an important role in the regulation of meiotic gene expression by DUET. In contrast, in yeast, *Drosophila* and mouse, DNA binding transcription factors have been identified as key regulators of meiotic gene expression, pointing to a DNA sequence based recognition mechanism [2,3,5]. DUET PHD finger shows similarity to animal proteins, and we identified DUET expressologs in other plant species, indicating that histone modifications could play a role in meiotic gene expression in other multicellular eukaryotes as well. Conversely, our work does not rule out that DUET is associated with DNA binding proteins that direct its recruitment to specific targets.

While most PHD fingers characterized to date have been shown to recognize H3K4me3 [14], we found that DUET PHD recognizes H3K4me2. In *Arabidopsis* and other eukaryotes, H3K4me2 accumulates at promoters and 5’ genic regions, but contrary to H3K4me3, does not correlate with active transcription. Rather, it is enriched on genes with tissue specific expression and with developmental functions [33,34,43–46]. Consistent with this notion, *TDM1* promoter has been found to be marked with H3K4me2 in seedlings [33,34], and we show that *TDM1* expression in young anthers is limited to meiotic cells.

### Developmental aspects of meiosis in plants

Previous studies showed that DUET is expressed specifically in anthers, suggesting a male specific function. We identified here two genes depending on DUET for expression during male meiosis, *TDM1* and *JAS*. Mutations in *TDM1* and *JAS* both result in male meiotic phenotypes. Moreover, we find that *TDM1*, whose expression in male meiosis directly depends on DUET, is not detected in female meiosis. Thus, a critical function of DUET might be to activate expression of male meiotic genes that contribute to sexual dimorphism particularly in the regulation of meiotic cell cycle transitions and spindle organization.

Our results also suggest a developmental function for H3K4me2 in *Arabidopsis* male reproduction. In *Drosophila* and *C*. *elegans*, H3K4me2 appears to be associated with male reproductive development. Mutations in *Drosophila* PHF7, which encodes an H3K4me2 PHD finger reading protein, result in loss of male germ cell identity [47]. In *C*. *elegans*, increase in H3K4me2 due to loss of an H3K4me2 demethylase, results in ectopic expression of spermatogenesis genes [48]. Thus, H3K4me2 patterns, and their interpretation by differentially expressed readers, may constitute a common feature of male reproductive development in multicellular eukaryotes.

## Materials and Methods

### Plant material

After 3 days at 4°C in the dark, seeds were germinated on MS media for 8–10 days. Seedlings were then transferred to soil and grown in a growth chamber under long day conditions. We obtained *ps1-4* (SAIL_1164_C09 in Col), *jas-3* (SAIL813_H03 in Col), and *tdm1-8* (SALK_124300 in Col) from the *Arabidopsis* Biological Ressource Center. We obtained *osd1-2* seeds (in Ler) from Raphael Mercier. The *duet* allele (in Ler) has been described previously [10].

### Pull down assays

For histone pull-downs, 5 μg of purified GST, GST-DUET-PHD and GST-BPTF-PHD recombinant proteins were incubated with 10 μg calf thymus histones (Amersham) in binding buffer (10 mM Tris-Cl pH 7.4, 300 mM NaCl, 5 mM EDTA, 1% Triton X-100, 0.1% BSA, 1mM phenylmethylsulphonyl fluoride (PMSF), and protease inhibitor) at 4°C overnight. Samples were then incubated with streptavidin beads for 1 h at 4°C. The beads were washed 5 times with 20 mM Tris-Cl pH 7.4, 150 mM NaCl, 1% Triton X-100, 10% glycerol 1 mM PMSF and protease inhibitor. Pull-downs were analyzed by western blot with core histone antibodies anti-H2A (1:1000), anti-H2B (1:4000), anti-H3 (1:1000) and anti-H4 (1:250). For histone peptide pull down assays, 1 μg of WT or mutated purified GST-DUET-PHD fusion protein were incubated with 1 μg biotinylated histone peptides (Upstate Millipore) were incubated together in binding buffer (50 mM Tris-Cl pH 7.5, 300 mM NaCl, 0.2% Triton X-100, 1 mM PMSF and protease inhibitor) at 4°C for 4 h followed by addition of streptavidin beads (Amersham) and incubating for 1h at 4°C. The beads were washed 4 times with 10 mM Tris-Cl pH 7.4, 0.05% Tween 20, 300 mM NaCl, 1 mM PMSF and protease inhibitor, and analyzed by western blot with an anti-GST antibody (Sigma).

### Antibody production

Two DNA fragments, corresponding to amino acid 303–340 and 651–704 respectively, were PCR amplified from DUET cDNA with AccuPrime Pfx DNA polymerase (Life Technologies) using primers DUET-AbA, DUET-AbB, and DUET-AbC and DUET-AbD respectively (primers are listed in S2 Table). The PCR fragments were joined by overlapping PCR, and cloned as a NdeI-HindIII fragment into pET28b as an N-terminal fusion to a 6x histidine (His) tag. The resulting construct was transformed into E.coli BL21 strain. The recombinant 6xHis-DUET protein was induced and purified using Ni-NTA agarose (QIAGEN). Rabbit and Rat polyclonal antiserums were produced against the His-DUET fusion protein, and were tested by western blot against the purified His-DUET protein.

### Constructs

DUET PHD finger was amplified by PCR using primers DUETphdBamHI and DUETphdEcoRI was cloned as a *BamHI*-*EcoRI* fragment into pGEX-3X to produce a N-terminal GST-DUET-PHD fusion. The resulting plasmid was mutated by site-directed mutagenesis with AccuPrime Pfx DNA polymerase (Life Technologies), and using primers listed in S2 Table. For complementation, a 3.8kb DUET fragment containing 1.3kb upstream the ATG and DUET coding sequence without stop codon, was PCR amplified using Accuprime Pfx DNA polymerase (Life Technologies), and cloned into pENTRY-D/TOPO according to manufacturer’s instruction (Life Technologies). The PHD finger was mutated by the same method and primers as described above. The resulting pENTRY clones were recombined in the binary vector pEARLEY302 using LR clonase according to manufacturer’s instructions (Invitrogen). For *pTDM1*::*NLS-GUS*, we amplified *TDM1* promoter from genomic Ler DNA, and recombined the fragment with pDONR41 by BP reaction (Initrogen). The resulting entry clone was cloned into destination vector pB7m34GW along with entry clones containing a 3xNLS and a GFP-GUS tag, respectively by LR reaction (Invitrogen). All clones were verified by sequencing and plants were transformed the floral dip method. Primary transformants (T1) were obtained by selecting seeds obtained from transformed plants on MS supplemented with 10 μg/ml BASTA. T1 plants were PCR genotyped for the construct and for the Ds transposon inserted in the *DUET* gene.

### Microarray and quantitative real-time PCR

For microarray analysis, about 150 0.5 mm-0.6 mm buds of 5–6 week old plants were collected in a 1.5 ml tube in liquid nitrogen. We purchased custom made *Arabidopsis* microarray from Agilent. The entire processing, including RNA extraction, procession and hybridization as well as the array analysis, was conducted by Genotypic (Bangalore) according to the Agilent’s instructions.

Anthers were dissected from 0.5 mm-0.6 mm buds of 5–6 week old plants and RNA was extracted with the plant RNeasy kit (QIAGEN), with on column DNA digestion with RNase free DNase (QIAGEN), according to the manufacturer’s instruction. 500 ng RNA was used for reverse-transcription using the Superscript III First-Strand Synthesis System (Invitrogen). Quantitative realtime PCR was performed in triplicates using Syber Green Master Mix (Life technologies) in an ABI Light Cycler. Dissociation curves were performed at the end of each run to confirm absence of genomic DNA contamination. Quantification of mRNA was calculated relative to *ACT11* from threshold points (C_t_ values) in the log-linear range of amplification plots using the 2- ΔC_t_ method.

### Cytology

Inflorescences with open flowers removed were fixed in 4% paraformaldehyde, 1x PBS, 0.1% Triton-X, for 2 hours under vacuum. Samples were rinsed at least 3 times with 1x PBS. For direct observation, anthers were dissected from meiotic buds in 2 μg/ml DAPI in 50% glycerol on a slide and squashed under a coverslip. For immunostaining, buds were digested with a mixture of cell wall degrading enzymes (0.3% cytohelicase, 0.3% pectolyase and 0.3% cellulose, all from Sigma) for 30 min at 37°C. Anthers were dissected in 1x PBS on a slide, squashed under a coverslip and plunged in liquid nitrogen. The coverslips were removed, and the slides left to dry. Cells were covered with a thin layer of 1% gelatin, 1% agarose, then left to dry, and cells were digested again as described above for 30 min at 37°C. Cell membranes were permeabilized in 1% Triton-X, 1x PBS for 30mn, and slides were rinsed 3 x 5 min in 1x PBS, 0.1% Triton-X. Immunostaining was performed as described [22]. Primary antibodies were used at 1:1000 (anti-ASY1), 1:50 (anti-tubulin, Harlan Sera-Lab MAS 077b), 1:100 (anti-H3K4me2, Abcam ab7766), 1:50 (anti-H3K9me2, Abcam ab1220), 1:5000 rabbit anti-DUET and 1:1000 rat anti-DUET. Anti-rabbit and anti-rat secondary antibodies conjugated with Alexa 488 or Alexa 594 fluor dyes (Life Technologies) were used at 1:100 to detect primary antibodies. For anti-DUET detection, an anti-rabbit or anti-rat HRP-conjugated secondary antibody was used at 1:100 for 2 hours at room temperature, and detection was performed using a tyramide amplification kit (Life Technologies) according to manufacturer’s instruction. Fluorescent images were acquired with a Zeiss Imager Z.1 microscope equipped with an ApoTome module and mounted with a Zeiss AxioCam HRM black and white camera. All fluorescent pictures are single section apotome images. Alexander staining was performed as described [20]. GUS staining was performed as described [49]. Young meiotic buds were opened and incubated without fixation in GUS staining solution for 4 days at 37°C. Detailled analysis of *pTDM1*::*NLS-GUS* expression pattern in prophase was done on segregating WT and *duet* T2 plants of 3 independent lines in which the construct segregated as a single locus. After 4 days incubating at 37°C in GUS staining solution, infloresences were fixed in 4% PFA, 1x PBS. Anthers were dissected out in 2 μg/ml DAPI solution and squashed to release and spread meiocytes. Images were acquired with a Zeiss Imager Z.2 mounted with a Zeiss AxioCam MRC color camera with differential interference contrast and with either an oil 63x or 100x Plan Apochromat DIC objective. All images were processed with Zeiss Axiovision software and figures were assembled with Adobe Photoshop CS5 extended.

### Chromatin immunoprecipitation

Chromatin immunoprecipitation (ChIP) was done using the Low Cell ChIP Kit from Diagenode according to manufacturer’s instructions. For each ChIP experiment, 200 inflorescences were collected from four to five week old WT Ler plants and crosslinked in Buffer A (0.4 M sucrose, 10 mM Tris pH 8, 1 mM EDTA, 1 mM PMSF, 1% formaldehyde). Chromatin was isolated according to manufacturer’s protocol. The suspended chromatin was sonicated for 15 cycles with low intensity on a Bioruptor UCD-200 sonicator, in which each cycle comprised of five sonication pulses of 15 seconds each with a one minute pause. The size of fragmented chromatin DNA was estimated to be between 0.5 and 1 kb by gel electrophoresis. Each sample was then split into two and 10% input was kept aside. Out of two one sample was incubated with rabbit anti-DUET antiserum and another with Rabbit IgG. Immunoprecipitations were performed according to manufacturer’s protocol and analysed on ABI 7900HT fast real time system using primers listed in S2 Table.

## Supporting Information

S1 Fig DUET specifically binds H3K4me2. (A) Histone peptide pull-down with the indicated peptides and followed by anti-GST western blot. (B) Alignment of DUET and characterized PHD fingers from animal proteins. (C) Phylogenetic tree made from the alignment in (b). DUET belongs to a group of H3K4me2/3 binding PHD fingers.
(TIF)

S2 Fig DUET is only expressed during diplotene during meiosis. (A) DUET immunostaining (green) on meiotic cells at interkinesis and telophase II. Only a single meiocyte at diplotene shows DUET signal. (B) DUET immunostaining on male meiotic squashes performed with a rat anti-DUET polyclonal serum.
(TIF)

S3 Fig Meiotic progression is delayed in *duet*. Frequencies of meiotic stages were established from anthers collected from buds measuring (A) 0.4–0.5 mm, (B) 0.5–0.6 mm, (C) 0.6–0.7 mm. The numbers indicate the total number of meiocytes that were counted.
(TIF)

S4 Fig Microscopic analysis of *duet* double mutants phenotypes. (A) Alexander staining of the indicated genetic backgrounds. (B) DAPI (blue) and DIC overlay images of meiosis I in the indicated backgrounds. Scale bar 10 μm. (C) DAPI (blue) and DIC overlay images of meiotic products in the indicated backgrounds. Scale bar 10 μm. (D) Meiotic progression analysis in the indicated backgrounds. The total number of meiocytes counted is indicated between brackets.
(TIF)

S5 Fig *pTDM1*::*NLS-GUS* expression during female meiosis. pTDM1::NLS-GUS expression during female meiosis. (A) late prophase, based on overall ovule and meiocytes morphology and (B) tetrad stage. Arrows point at meiocytes. Scale bar 10 μm.
(TIF)

S6 Fig Identification of DUET expressologs suggests conserved function in plants. Patterns of expression of DUET homologues in the indicated plant species.
(TIF)

S1 Table Profiling of gene expression during meiosis in wild type and *duet*. Sheet 1 –genes showing 2-fold or greater differential expression in *duet*. Sheet 2 –set of meiotically preferred genes from Libeau et al., 2011 showing differential expression in *duet*. Sheet 3 –qPCR validation of a subset of differentially expressed meiotically preferred genes. Sheet 4 –GO term analysis for downregulated meiotic genes. Sheet 5 –GO term analysis for upregulated meiotic genes.
(XLSX)

S2 Table List of primers used in the study. (XLSX)
