# Supplementary figures and images for: The Chromatin Protein DUET/MMD1 Controls Expression of the Meiotic Gene TDM1 during Male Meiosis in Arabidopsis
Source: PLoS Genet. 2015 Sep 8;11(9):e1005396. doi: 10.1371/journal.pgen.1005396 (PMC4562639; doi:10.1371/journal.pgen.1005396)

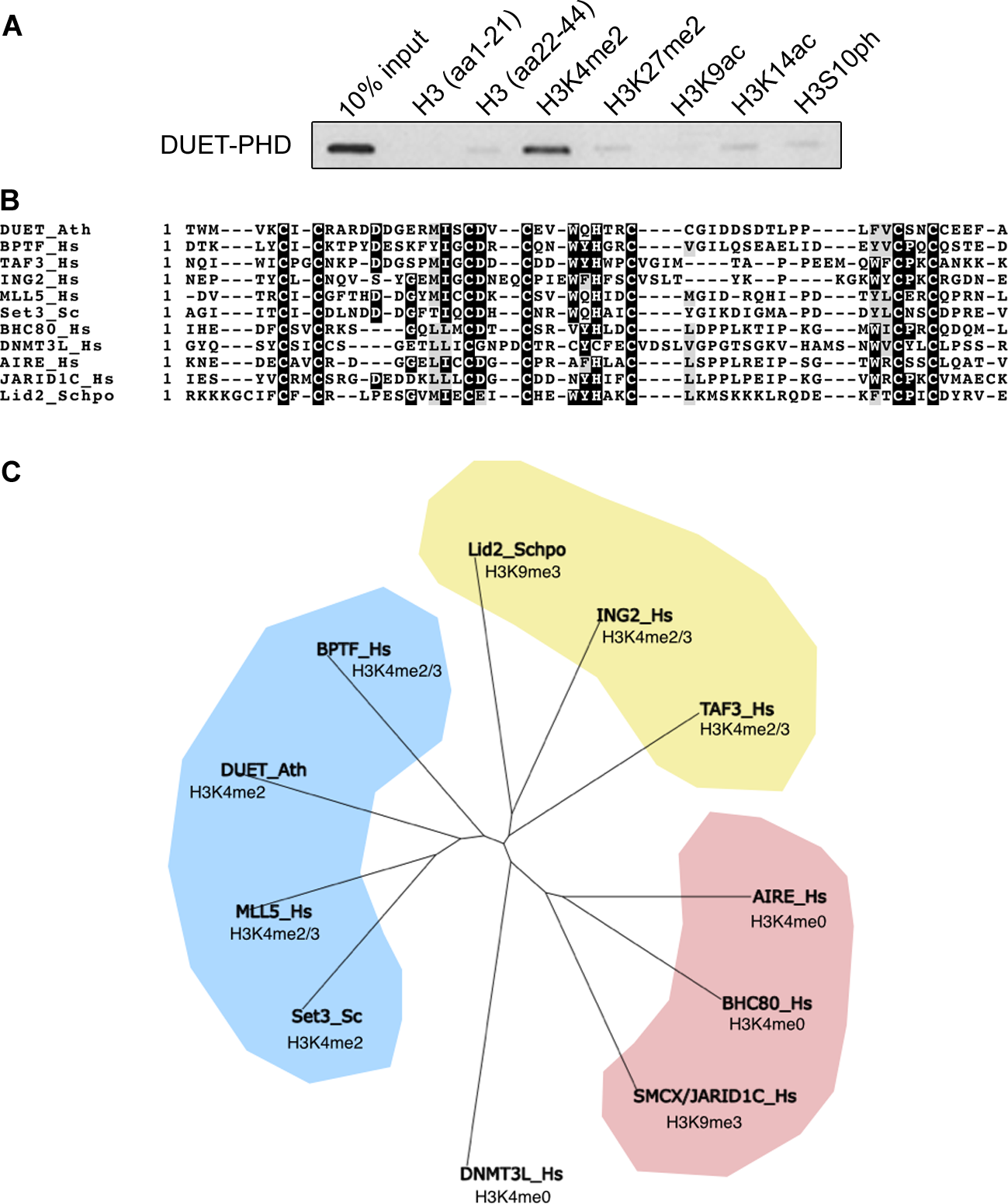

Supplement: S1 Fig — (A) Histone peptide pull-down with the indicated peptides and followed by anti-GST western blot. (B) Alignment of DUET and characterized PHD fingers from animal proteins. (C) Phylogenetic tree made from the alignment in (b). DUET belongs to a group of H3K4me2/3 binding PHD fingers. (TIF) [file pgen.1005396.s001.tif]

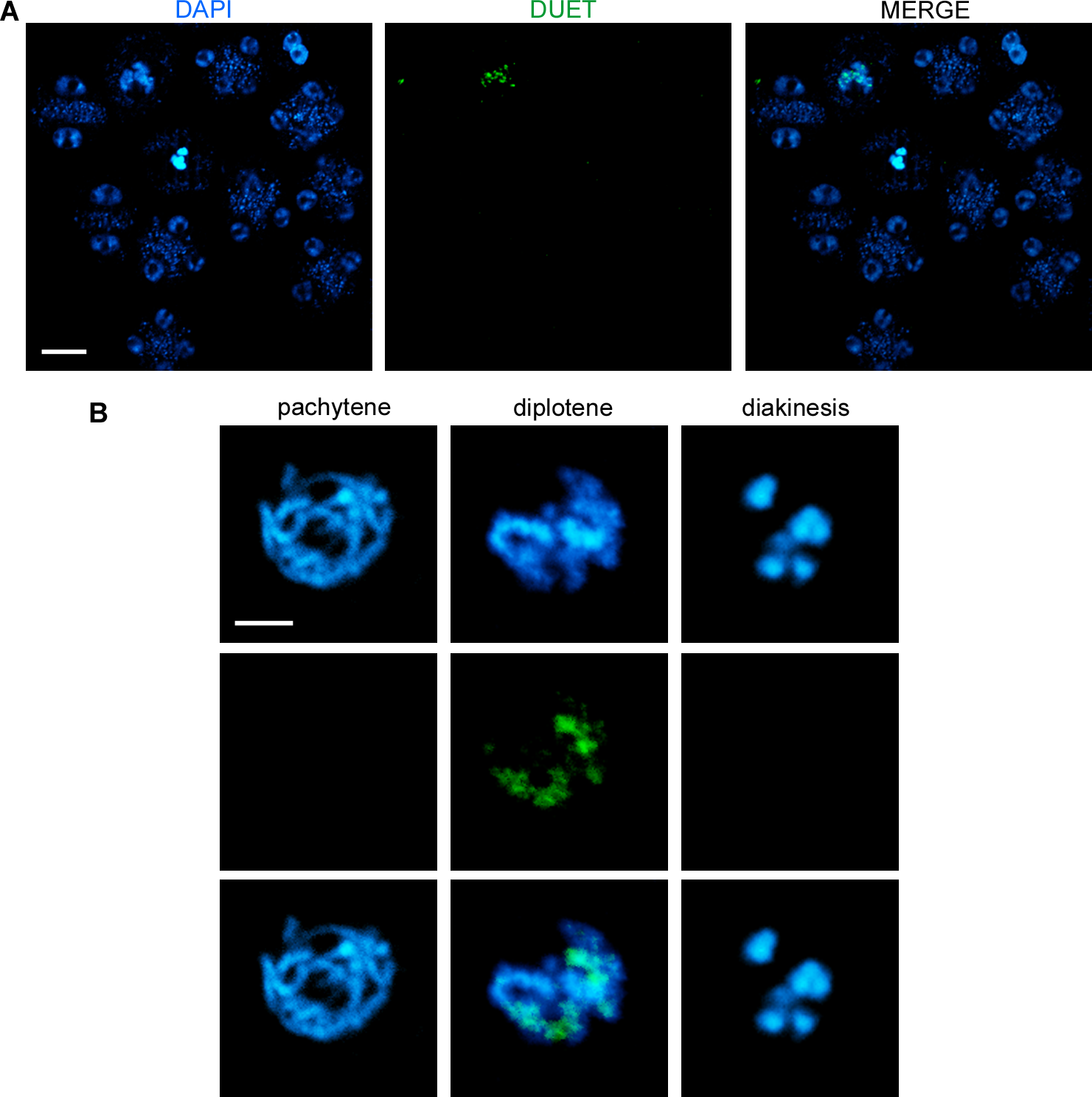

Supplement: S2 Fig — (A) DUET immunostaining (green) on meiotic cells at interkinesis and telophase II. Only a single meiocyte at diplotene shows DUET signal. (B) DUET immunostaining on male meiotic squashes performed with a rat anti-DUET polyclonal serum. (TIF) [file pgen.1005396.s002.tif]

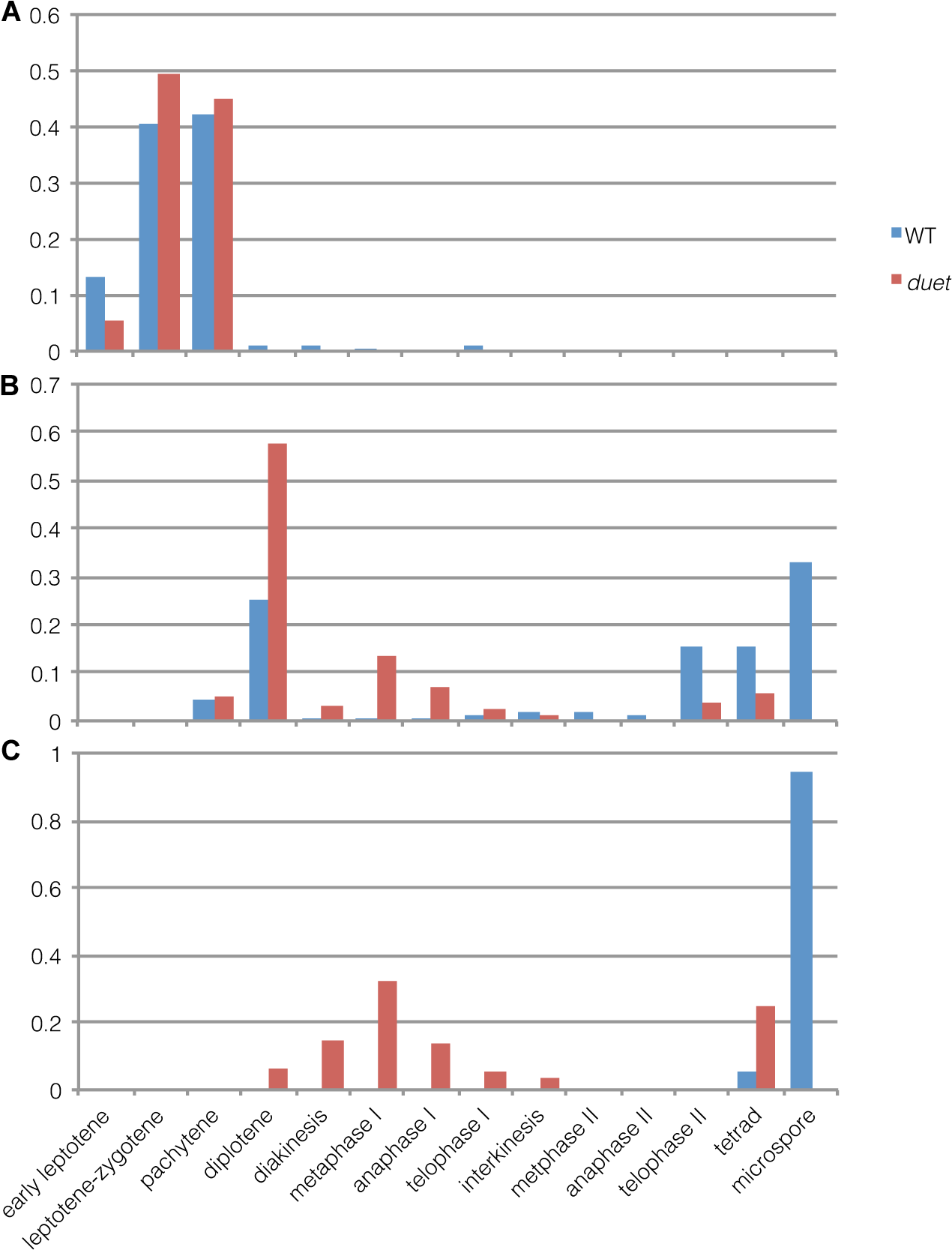

Supplement: S3 Fig — Frequencies of meiotic stages were established from anthers collected from buds measuring (A) 0.4–0.5 mm, (B) 0.5–0.6 mm, (C) 0.6–0.7 mm. The numbers indicate the total number of meiocytes that were counted. (TIF) [file pgen.1005396.s003.tif]

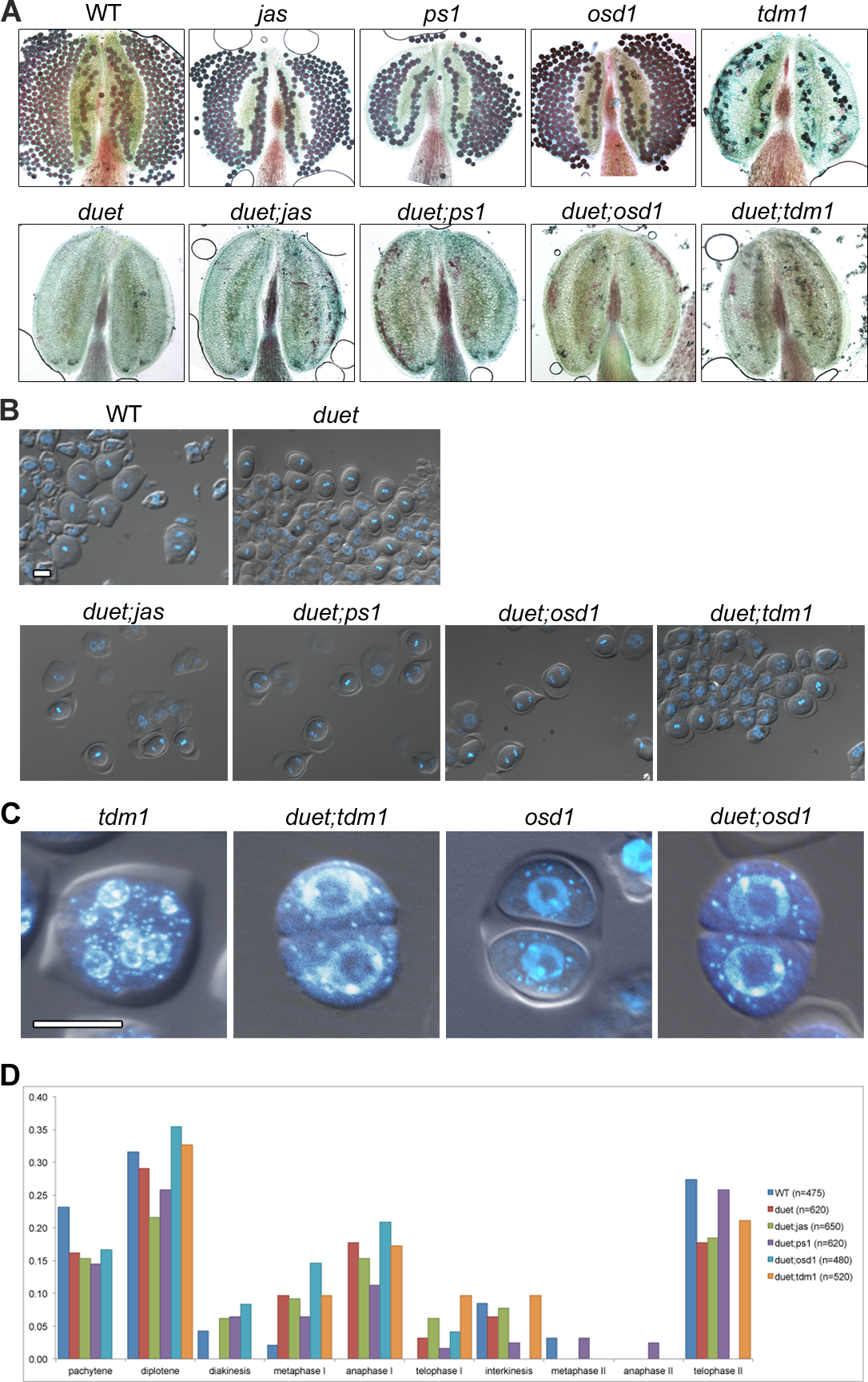

Supplement: S4 Fig — (A) Alexander staining of the indicated genetic backgrounds. (B) DAPI (blue) and DIC overlay images of meiosis I in the indicated backgrounds. Scale bar 10 μm. (C) DAPI (blue) and DIC overlay images of meiotic products in the indicated backgrounds. Scale bar 10 μm. (D) Meiotic progression analysis in the indicated backgrounds. The total number of meiocytes counted is indicated between brackets. (TIF) [file pgen.1005396.s004.tif]

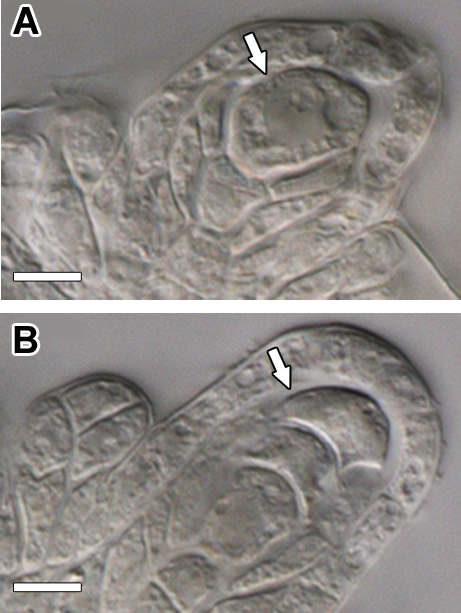

Supplement: S5 Fig — pTDM1::NLS-GUS expression during female meiosis. (A) late prophase, based on overall ovule and meiocytes morphology and (B) tetrad stage. Arrows point at meiocytes. Scale bar 10 μm. (TIF) [file pgen.1005396.s005.tif]

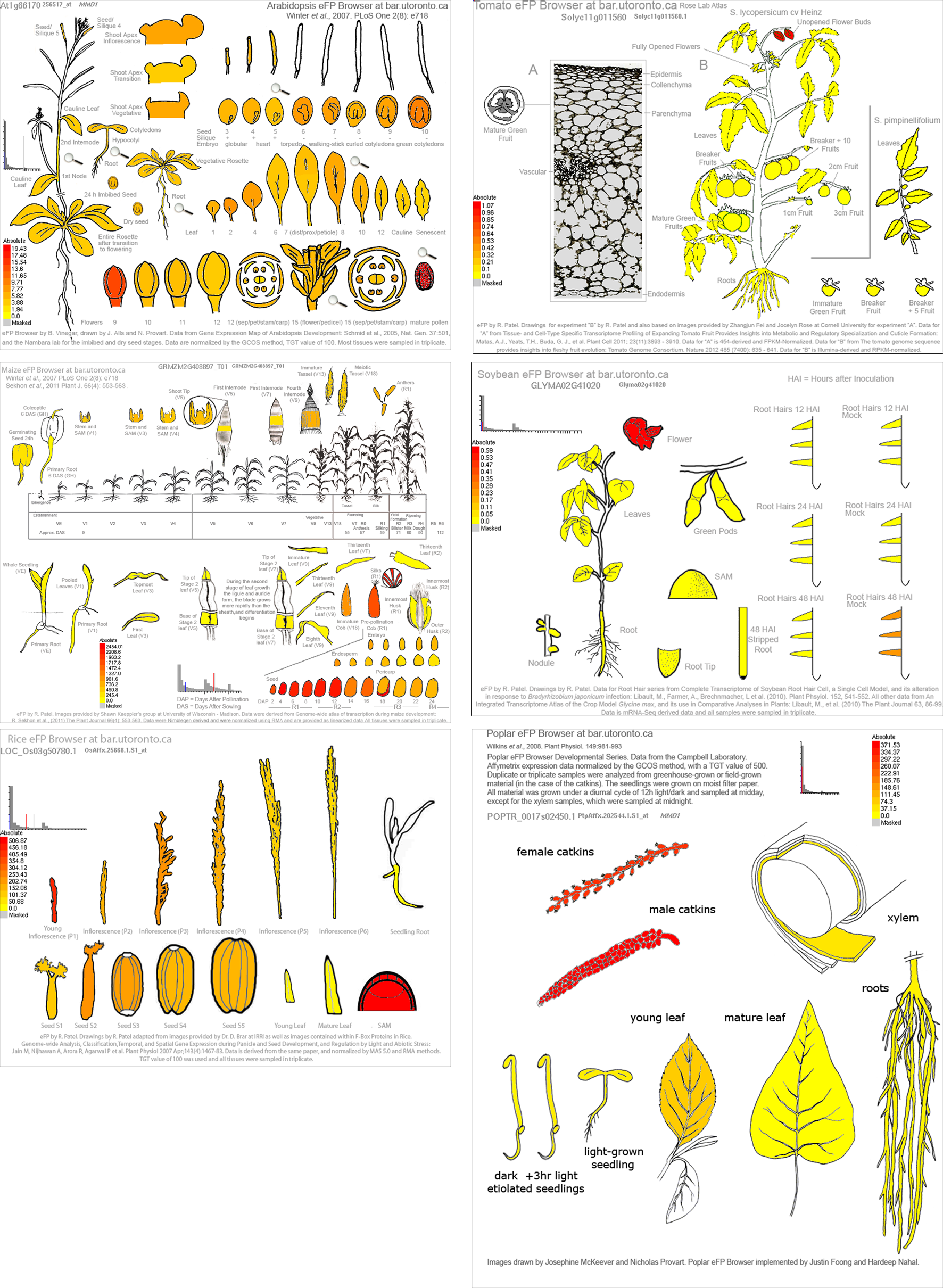

Supplement: S6 Fig — Patterns of expression of DUET homologues in the indicated plant species. (TIF) [file pgen.1005396.s006.tif]
